# Performance indicators for initial regional medical response to major incidents: a possible quality control tool

**DOI:** 10.1186/1757-7241-20-81

**Published:** 2012-12-17

**Authors:** Heléne Nilsson, Tore Vikström, Carl-Oscar Jonson

**Affiliations:** 1 Department of Clinical and Experimental Medicine, Centre for Teaching and Research in Disaster Medicine and Traumatology (KMC), Linköping University Hospital, Faculty of Health Sciences Linköping, S-581 83, Linköping, Sweden

**Keywords:** Emergency response, Disaster management, Casualty incident, Quality measurement, Resource management

## Abstract

**Background:**

Timely decisions concerning mobilization and allocation of resources and distribution of casualties are crucial in medical management of major incidents. The aim of this study was to evaluate documented initial regional medical responses to major incidents by applying a set of 11 measurable performance indicators for regional medical command and control and test the feasibility of the indicators.

**Methods:**

Retrospective data were collected from documentation from regional medical command and control at major incidents that occurred in two Swedish County Councils. Each incident was assigned to one of nine different categories and 11 measurable performance indicators for initial regional medical command and control were systematically applied. Two-way analysis of variance with one observation per cell was used for statistical analysis and the post hoc Tukey test was used for pairwise comparisons.

**Results:**

The set of indicators for regional medical command and control could be applied in 102 of the130 major incidents (78%), but 36 incidents had to be excluded due to incomplete documentation. The indicators were not applicable as a set for 28 incidents (21.5%) due to different characteristics and time frames. Based on the indicators studied in 66 major incidents, the results demonstrate that the regional medical management performed according to the standard in the early phases (1–10 min after alert), but there were weaknesses in the secondary phase (10–30 min after alert). The significantly lowest scores were found for Indicator 8 (formulate general guidelines for response) and Indicator 10 (decide whether or not resources in own organization are adequate).

**Conclusions:**

Measurable performance indicators for regional medical command and control can be applied to incidents that directly or indirectly involve casualties provided there is sufficient documentation available. Measurable performance indicators can enhance follow- up and be used as a structured quality control tool as well as constitute measurable parts of a nationally based follow-up system for major incidents. Additional indicators need to be developed for hospital-related incidents such as interference with hospital infrastructure.

## Background

Despite the fact that lessons learned from major incidents and disasters in the past have resulted in many improvements, shortcomings still exist [1-4]. We know from incidents involving casualties that a rapid response, accurate triage and controlled evacuation and distribution of casualties are important factors that influence the outcome for the victims [5-8]. Other studies have shown that regional coordination of medical resources improves patient flow, reduces time to definitive care and thereby improves patient outcome [9]. Even though there are differences between countries, this level of management is often referred to as strategic management, gold level or regional medical command and control [5,7,10-14].

**Figure 1:**
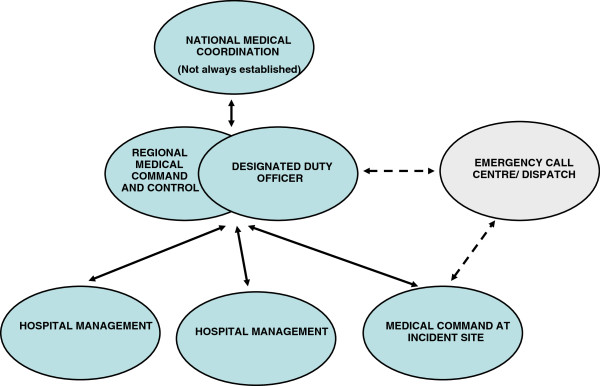
Schematic model of Swedish medical incident command and control system at major incidents.

The Swedish National Board of Health and Welfare (NBHW) has issued regulations for how the medical management of major incidents and disasters should be carried out [15]. The term major incident is used in the Swedish system as a generic response term for different types of events including risk and threat situations, e.g. transportation accidents, spread of hazardous material, infrastructure disruptions, armed aggression, and psychosocial impact on society as a result of traumatic events. The decision to declare a major incident is made by a designated duty officer (DDO) at the regional level and is influenced by the type and magnitude of the incident, and what potential impact the event might have on health care [11,14,15] (Figure 1).

The indicators used in this study are derived from a national concept and process modelling of management in major incidents and disasters, conducted by the NBHW and have been described in two previous studies [16,17]. The measurable performance indicators extracted from this process have been used for many years in Swedish disaster management training. In addition, the same indicators have been used in an international study and as an evaluation tool in full-scale exercises [18-21].

Indicators for quality control are well-established within most areas of health care, but there is still a need for their further development and implementation in the field of disaster medicine. One way to address these issues is to study if performance indicators for initial regional medical command and control can be used as a quality control tool, and thereby could be included in regional medical response plans and constitute measurable parts of a nationally based follow-up system for major incidents.

The aim of this study was to evaluate documented initial regional medical response to major incidents by applying a set of 11 measurable performance indicators for regional medical command and control and test the feasibility of the indicators.

## Methods

This was a retrospective observational study of 130 major incidents occurring in two County Councils in Sweden between 2006 and 2009. Data in this study were collected from two County Councils who had fully implemented the national medical incident command and control system The personnel acting as DDO in these two County Councils are similar in terms of competencies and background. They have a clear regional mandate to declare major incidents and to take immediate medical decisions over all regional medical resources [22,23] (Figure 1). The two County Councils are located in one of Sweden’s largest metropolitan areas after Stockholm, Gothenburg and Malmö, with approximately 699 000 inhabitants living in urban and rural areas.

All available documentation from the regional command and control for 130 incidents, all declared as a major incident, were studied with regard to type of incident, staff resources required for regional management, and how long the regional management body remained active. All incidents studied were classified into the following nine categories: accidents; fires; interferences with hospital infrastructure; chemical, biological, radiological, nuclear, and explosive (CBRNE) events; infectious events; weather alerts; support asked from another region; and incidents abroad with a regional impact (Table 1).

**Table 1:** Classification of 130 major incidents that occurred in two Swedish County Council regions, 2006–2009

**Classification**	**Distribution (*n *= 130)**
Accidents	48
Threats	37
Fires	23
Interference with hospital infrastructure	15
CBRNE events	2
Infectious events	2
Weather alert	1
Support to other region	1
Incident abroad with a regional impact	1

**Figure 2:**
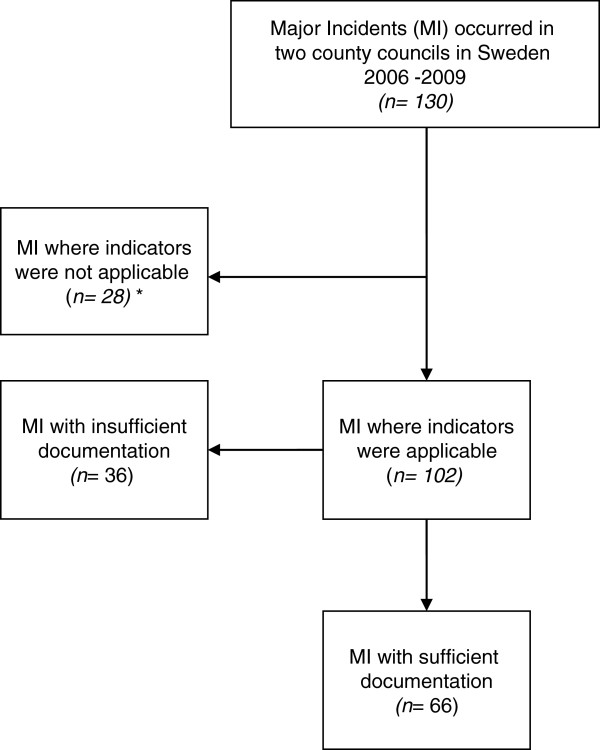
**Study case flow.** *Interruptions in hospital infrastructure, infectious events, incidents abroad, regional support asked from other region, weather alerts.

A set of 11 previously developed measurable performance indicators for assessing initial regional medical command and control were systematically applied (Figure 2) [16,17]. Each indicator was given a score of 0, 1 or 2 points; 0 = objective was not met at all, 1 = objective met but not within the stipulated time frame, 2 = objective completely met within stipulated time frame. The average score for each indicator was calculated (Table 2).

**Table 2:** Mean scores (0–2) for 11 performance indicators of initial regional medical command and control in 66 major incidents

**Performance indicator (standard within *x *minutes from alert)**	**Mean score (0–2)**
1. Declaration of major incident (1 min)	1.48
2. Decision on level of alert for staff (3 min)	1.41
3. Decision on sending additional resources to scene (3 min)	1.32
4. Decision on receiving hospitals (5 min)	1.63
5. Establishing contact with incident officers at scene (10 min)	1.53
6. Decision on preliminary referrals (10 min)	0.86
7. First information to media (15 min)	0.75
8. Formulate general guidelines for response (15 min)	0.18
9. Ensuring that there is adequate information for decision on referrals (20 min)	0.93
10. Assessment if resources in own organization are adequate (30 min)	0.03
11. Notify decision on referrals to receiving hospitals (40 min)	0.97

### Statistics

A two-way analysis of variance with one observation per cell and the post hoc Tukey test for pairwise comparisons were used. A *p* value <0.05 was considered significant. Minitab version 16 (Minitab Inc®, http://www.minitab.com) was used for the statistical calculations.

## Results

### Descriptive results

During the period from 2006 to 2009, 130 incidents were declared as major incidents. Regional medical command and control was established in various types of incidents (Table 1). Approximately 1229 casualties (range 3–135/incident) were directly involved in 102 major incidents (78%) classified as accidents, fires, threats and CBRNE events. In 35 major incidents (27%), casualties were distributed to more than one hospital and in 15 major incidents (11.5%) one or more hospitals activated their hospital disaster plan.

**Figure 3:**
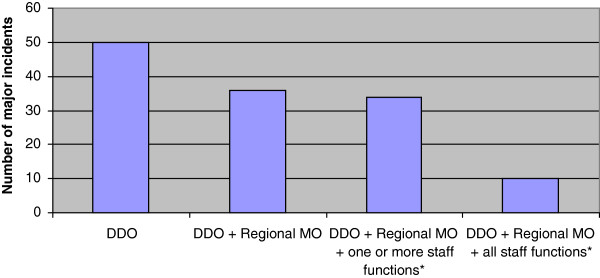
**Extent of regional medical command and control in 130 major incidents.** DDO, designated duty officer. Regional MO, medical officer at regional level. Staff functions; experts on public information and communication, psychological trauma support, hospital infrastructure and other administrative or medical support.

**Figure 4:**
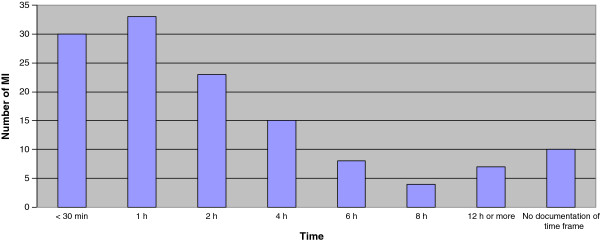
**Length of time regional medical command and control were active in 130 major incidents. Median value = 60 minutes.** MI= major incident.

Regional medical command and control was established by the DDO alone in 50 of the 130 major incidents (38%), and in 36 major incidents (28%), a specific regional medical officer (physician) was also alerted. In 34 major incidents (26%), one or more staff positions were called to support the management, such as experts on public information and communication, psychological trauma support or other experts in specific medical or management fields. A more comprehensive regional medical management group consisting of an increased number of staff positions was established in 10 major incidents (7%) (Figure 3). In 98 major incidents (75%), the regional medical command and control was active for 4 h or less (median time 60 min) (Figure 4). The documentation for 36 major incidents was incomplete for accurate evaluation.

### Performance indicators

The indicators were not applicable as a set in 28 incidents (21.5%) due to different characteristics and time frames. These incidents involved interference with hospital infrastructure requiring regional support (power failure, IT disturbance, phone interruptions), an incident occurring in another region (evacuation of in-hospital patients), an incident abroad having a regional impact (evacuation of Swedes from Lebanon), weather alerts (storms), and infectious events (suspected water contamination, mass vaccination during the H1N1 flu pandemic). The 11 measurable performance indicators assessing the initial regional medical command and control were applied in 102 major incidents (78%) in the following categories: accidents, fires, threats and CBRNE events. Thirty-six of the 102 major incidents were excluded due to incomplete documentation (Figure 2).

A total of 726 measurable performance indicators were collected from 66 major incidents involving accidents, fires, threats and CBRNE events. Four hundred and forty-six indicators (61%) were met completely or partly, and in 280 indicators (38%), the objective was not met at all.

The mean score for each performance indicator ranged from 0.03 to 1.63 out of a maximum score of 2 (Table 2). Indicator 4 and Indicator 5 had the highest mean values. Indicator 8 and Indicator 10 had the lowest mean values.

**Figure 5:**
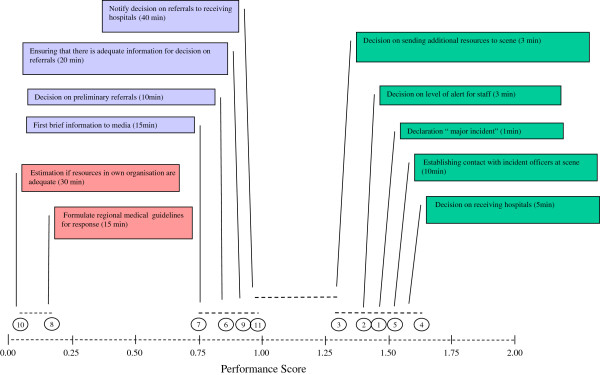
**Comparison of scores from 11 different performance indicators in 66 MI (Table 2).** The mean values of the 11 indicators are on the baseline. The numbers of each performance indicator are circled (Table 2). Numbers that lie below the same horizontal line are not significantly different from each other. For example: Scores from Indicators 8 and 10 differ significantly from the rest of the indicators (1, 2, 3, 4, 5, 6, 7, 9, and 11).

Comparison of the results shows that performance indicators measuring decisions in the early phase of an incident (1–10 min after alert) had a significantly higher mean score than indicators measuring decisions in the secondary phase (e.g. 10–40 min after alert) (*p*<0.05). Performance Indicator 8 (formulate general guidelines for response) and Indicator 10 (decide if resources in own organization are adequate) differed significantly from Indicators 1, 2, 3, 4, 5, 6, 7, 9, and 11 (*p*<0.05). There was no significant difference between Indicator 3 (decision on additional resources to the scene) and Indicator 11 (notify decision for referral to receiving hospital) (Figure 5) [24].

## Discussion

This was a study of the initial regional response to major incidents built on traceability and in compliance with a specific protocol of 11 measurable performance indicators. In the final systematic review of 66 major incidents, all performance indicators could be applied and used for assessing the regional medical response. The difficulty with the collection of data was mostly due to the lack of documentation. A decision may have been made or considered but unfortunately never documented. A prerequisite is that the indicators are known and accepted by the organizations and that the documentation is detailed enough.

Initial actions taken (often by the DDO and a regional medical officer–physician) within the first 10 min, such as declare major incident, alert to receiving hospitals and establish contact with medical command on scene, were often done correctly and on time. Decisions made after 10 min, usually concerning the distribution of casualties, were often somewhat delayed. The reason for this could be that the DDO had to wait for reports and additional information from the medical incident command on the scene. Our study of the regional documentation files revealed that the prehospital reports were sometimes not sent at all, or not according to standard operating procedures (e.g. first report within 3 min and a second verifying report within 10 min of arrival on the scene) resulting in the DDO being forced to obtain the information via the emergency dispatch centre instead. This unnecessary procedure could be one of the factors affecting the ability to make decisions at the right time.

Delays in decisions concerning distribution of victims might not be fatal in a minor incident, but can be crucial in situations with a more rapid course of events with a risk of overloading the nearest hospital [6,25-27]. Several studies on incidents involving casualties show that effective casualty distribution plays a vital role in disaster management, especially if the incident occurs in a rural area where resources are limited [5,7,27,28].

Another well-known truism for disasters is that the hospital or health care facility closest to the incident site will be the one most significantly affected by a large number of casualties and when timely notification is lacking, the hospitals will need to respond with the resources on hand. In addition, not seriously injured casualties self-refer to the hospital they are most comfortable with. [12,29,30]. In this study, we found that the casualties were distributed to more than one hospital according to a distribution key delivered by the regional medical command and control in 35 major incidents. Therefore, execution of a planned and timely distribution from a regional overall health care perspective can be beneficial, thus reducing the impact on daily activities and patient surge following an unnecessary activation of a local hospital disaster plan [25].

The regional command and control alerted a neighbouring county on one occasion only. The reason could be that the major incidents were not of such magnitude or there was no other reason to request resources from another region. It may also be that this decision was considered, but was not documented in the log file. However, there may also be a fundamental barrier such that neighbouring counties are only alerted when resources begin to run out. In a minor incident, it is probably enough to distribute casualties to the hospitals within one county, but in a major incident involving a large number of casualties, early contact with neighbouring counties can be crucial, particularly when higher levels of trauma care are required [25]. The significantly low mean score for Indicator 11 suggests that the importance of an early alert and establishment of cooperation between County Councils needs to be stressed even more in education and training.

Another weakness observed in regional management was the absence of formulating guidelines for response, or in other words, taking a set of objectives and designing a strategic plan to mitigate any consequences of the incident. In simulation exercises, this type of strategic decision making has also been shown to be one aspect of regional management that needs to be improved [20]. However, such a plan might have been considered but was not recorded in the log file. This may be very difficult to achieve in the early intensive phase of a major incident, but could have vital consequences for subsequent direction and evolution and could influence patient outcome. This emphasizes the need for more preparatory training and education for staff involved in strategic and goal-oriented decision making at the regional level of medical command.

The study demonstrates that a DDO at the regional level of health care has to deal with several types of major incidents, all with different characteristics and time lines. The experience from Khorram-Manesh et al. [14,31] showed that incidents that interfere with the hospital infrastructure such as power or IT system failure also have an impact on regional preparedness and that the frequency of these types of hospital-related incidents has increased. The performance indicators used in this study were not applicable to these types of incidents. Even though an individual indicator (e.g. declare major incident) could be applied, most of the other indicators would have to be adjusted with regard to other objectives and time standards.

This study shows that in 77% of incidents, the regional medical command and control was active for 4 h or less and most incidents were handled by the DDO, a regional medical officer (physician) with one or two staff functions. This emphasizes the need to pay special attention to the important time perspectives when building up a regional response organization with regard to response time and the medical competencies needed to handle all types of major incidents.

### Limitations of the study

Our study has some limitations due to its retrospective design and the lack of coherent incident documentation. Although correct and relevant documentation of incident management is a prerequisite for evaluation and follow-up, lack of documentation is a common problem in disaster evaluation studies [32,33]. This study was also limited to major incidents with sufficient documentation and therefore we cannot rule out the risk of selection bias. However, what we do know is that the category distribution of the dropouts does not differ from the rest and the indicators could have been applied if documentation had been more comprehensive. In the future, the implementation of digital support systems that can provide real-time data, capture information and share it along the chain of medical command might increase the efficiency and resource management and also facilitate follow-up at all levels [34,35].

In summary, in order to implement an effective quality control of response to major incidents, specific standards for the regional medical response needs to be set. The quality control process of regional medical response at major incidents must be ongoing to ensure effective response and to early detect deficiencies that continuously leads to quality improvements.

Measurable performance indicators enable a structured and objective evaluation of incident management, can identify areas for improvement, and could facilitate a systematic follow-up of major incidents. Further prospective studies are needed to examine if the time taken for regional decisions about distribution of casualties correlates with patient time at the scene, time to definitive care and patient outcome.

## Conclusions

Measurable performance indicators for initial regional medical response are feasible to use as a quality control tool provided that there is sufficient documentation available. The indicators can be applied on major incidents that directly or indirectly involve casualties and could constitute measurable parts of regional and national follow-up systems. Modification of the present indicators and additional indicators might be needed to assess hospital-related incidents. Future introduction of digital information and support systems for incident management could provide more accurate and coherent documentation to support follow-up of major incidents at all levels.

## Competing interests

This work was performed by the Centre of Teaching in Disaster Medicine and Traumatology (KMC), Linköping, Sweden. The authors declare that they have no competing interests.

## Authors' contributions

HN was involved in the study design, data collection, analysis, and manuscript writing. COJ was involved in the analysis and manuscript writing. TV was involved in the study design and contributed to the finalization of the manuscript. All authors read and approved the final version of the manuscript.
